# Teenage Pregnancy: A Socially Inflicted Health Hazard

**DOI:** 10.4103/0970-0218.55289

**Published:** 2009-07

**Authors:** Bratati Banerjee, GK Pandey, Debashis Dutt, Bhaswati Sengupta, Maitrayei Mondal, Sila Deb

**Affiliations:** Department of Community Medicine, Maulana Azad Medical College, New Delhi, India; 1 Department of Epidemiology, All India Institute of Hygiene and Public Health, Kolkata, India; 2 Department of Public Health Administration, All India Institute of Hygiene and Public Health, Kolkata, India

**Keywords:** Anemia, complications, low birth weight, preterm delivery, teen pregnancy

## Abstract

**Background:**

Early marriage and confinement are contributing factors to high maternal and perinatal mortality and morbidity.

**Objective:**

To assess the magnitude of the problem of teenage pregnancy and its complications.

**Materials and Methods:**

A hospital-based cohort study was undertaken over 4 months among women admitted to a rural hospital in West Bengal. The study cohort comprised of teenage mothers between 15-19 years old and a control cohort of mothers between 20-24 years old. Data included demographic variables, available medical records, and complications viz. anemia, preterm delivery, and low birth weight. Anemia was defined as a hemoglobin level below 10 gm% during the last trimester of pregnancy, preterm delivery was defined as occurring within 37 weeks of gestation, and low birth weight was defined as babies weighing less than 2500 grams at birth.

**Result:**

Teenage pregnancy comprised 24.17% of total pregnancies occurring in the hospital during the study period. The study group had 58 subjects and the control group had 91 subjects. The prevalence of anemia was significantly higher (*P*<0.05) in the women in the teenage group (62.96%) than in the women in the control group (43.59%). However, severe anemia with a hemoglobin level below 8 gm% was only found in the control group. Preterm delivery occurred significantly more (*P*<0.001) in the study group (51.72%) than in the control group (25.88%). The incidence of low birth weight was significantly higher (*P*<0.0001) among the group of teenagers (65.52%) than among the women in the control group (26.37%). Not a single newborn was above 3 kg in the study group, while none were below 1.5 kg in the control group. The mean birth weight was 2.36 kg in the study group and 2.74 kg in the control group; the difference was strongly significant (*P*<0.001).

**Conclusion:**

The study shows that anemia, preterm delivery, and low birth weight were more prevalent among teenagers than among women who were 20-24 years old. This indicates the need for enhancing family welfare measures to delay the age at first pregnancy, thereby reducing the multiple complications that may occur in the young mother and her newborn baby.

## Introduction

Health, in addition to its biomedical determinants, is influenced by many social and cultural factors. This influence is often negative with a resultant increase in the number of social hazards, which finally aggravate the already poor health status of the developing societies. One such social hazard of serious consequences on the nation as a whole is pregnancy in an adolescent girl, who herself is yet to attain her full growth potential.(1)

The transition from childhood to adulthood may be referred to as ‘adolescence’ or ‘teenage’, which has been defined by the World Health Organization as the period between 10-19 years.(2) This is the period when structural, functional, and psychosocial developments occur in a child to prepare her for assuming the responsibility of motherhood.

Child marriage and early confinement is a long established custom in India, with poverty and ignorance magnifying the problem.(3) In our country, teenage pregnancies after marriage, in contrast to unwed pregnancies in developed countries, have social approval but have an adverse impact on maternal mortality and perinatal morbidity. Pregnancy in very young women is generally considered to be a very high risk event, because teenage girls are physically and psychologically immature for reproduction. In addition, there are some extrinsic factors such as inadequate prenatal care, illiteracy, and poor socio-economic conditions that affect the outcome of pregnancy in teenage girls.(4–6) Several medical complications like preterm birth, poor maternal weight gain, pregnancy-induced hypertension, anemia, and sexually transmitted diseases are strongly associated with teenage pregnancy.(2) It also adversely affects the status of women. Preserving the health of women has been given high priority in the Reproductive and Child Health Program.(7) Knowing the burden of pregnancy in teenagers will go a long way in advocacy and devising appropriate intervention measures.

The state of west Bengal shows a similar dismal picture in having a high prevalence of teenage pregnancy along with its complications.(8–10) This study was carried out in a rural area of West Bengal with the objective of assessing the magnitude of teenage pregnancy and the associated common complications namely anemia, preterm delivery, and low birth weight.

## Materials and Methods

A cross sectional, institution-based study was undertaken over a period of 4 months in a rural hospital in the South 24 Parganas district in West Bengal. The study group consisted of teenage mothers between 15-19 years old who were admitted to and delivered in the hospital during the study period, who were compared with a control group of mothers between 20-24 years old who were admitted to and delivered in the same hospital during the same period. This group was chosen as the control group because adverse outcomes are expected to be least in this group.(11) Women who were referred to other hospitals were excluded from the study, as there was no scope for follow-up.

Clearance for the study was taken from the Ethical Committee of the Institute from where it was conducted and approval was taken from the study hospital. It was a descriptive study and no intervention was done. The data collected using a pre tested, semi-structured schedule was part of a routine process of obtaining the patient's medical history and an examination. The medical history included demographic, socio-economic, marital, reproductive, investigation and treatment history, a review of available medical records, and an examination of the mother and infant to determine the pregnancy outcome. Hence, the study was conducted based on implied consent. However, the purpose of the study was explained to the subjects and no difference was made between minor and adult subjects. Primary data was collected by interviewing the women or their guardians and by examination of the mothers and their newborns. Medical records available with the women and records and registers of the hospital were used for collecting secondary data.

In this study, three common complications of pregnancy were analysed: anemia, preterm delivery, and low birth weight. Anemia was defined as a hemoglobin level below 10 gm% in the last trimester of pregnancy, preterm delivery was defined as occurring within 37 weeks of gestation, and low birth weight was defined as babies weighing less than 2500 grams at birth.(11)

## Results

The number of total confinements in the hospital during the 4-month period was 302, of which 73 (24.17%) subjects were between 15-19 years old and 109 (36.09%) subjects were between 20-24 years old. Of these, 58 (79.45%) teenagers and 91 (83.49%) 20-24 year old women delivered in the study hospital and therefore were eligible to take part in the study comprising the study group and control group, respectively. Thus, the referral rate was 20.55% and 16.51% in the study and control groups, respectively; the difference was not significant. Teenage pregnancy comprised 24.17% of the total pregnancies that occurred in the hospital during the study period.

All the subjects were married. Both the groups had similar socioeconomic status. The majority were primipara - 90% and 82% in the study and control groups, respectively. As the number of second and third parous women was too small, separate analysis was not done for parity. All of them had started antenatal care from the hospital OPD late in the 1^st^ trimester or early in the 2^nd^ trimester and had 3 or more antenatal check-ups, as recommended under the RCH program. The quality of antenatal care delivered from the same institution was similar in the two groups. No significant difference was observed between the two groups in respect to their health and nutritional status other than the parameters under study.

All the 58 teenagers had documented records for last menstrual period, as compared with 85 of the 91 women in the control group. Results of the hemoglobin estimation were known in 54 teenagers and 39 women in the control group. The prevalence of anemia [Table 1] was significantly more (*P*<0.05) in the teenage group (62.96%) in comparison with the control group (43.59%). Preterm delivery was seen to occur more commonly in the study group (51.72%) than in the control group (25.88%) and this difference was significant at *P*<0.001. The incidence of low birth weight was observed to be significantly higher (*P*<0.0001) among the teenage mothers (65.52%) than among the mothers in the control group (26.37%).

**Table 1:** Distribution of subjects according to complications of pregnancy

Complications	Study cohorts			Control cohorts			Level of significance	
			
	Total pregnancies	Complications		Total pregnancies	Complications		Z value	*P* value
						
		No.	%		No.	%		
Anemia	54	34	62.96	39	17	43.59	1.85	<0.05
Preterm delivery	58	30	51.72	85	22	25.88	3.15	<0.001
Low birth weight	58	38	65.52	91	24	26.37	4.72	<0.0001

Though the prevalence of anemia was higher (*P* < 0.05) in the study group, severe anemia with hemoglobin levels below 8 gm% were found only in the control group [Table 2].

**Table 2:** Distribution of subjects according to hemoglobin status

Hemoglobin level (gm %)	Study group		Control group	
		
	No.	%	No.	%
7-7.9	0	-	2	5.13
8-8.9	5	9.26	3	7.69
9-9.9	29	53.70	12	30.77
≥10	20	37.04	22	56.41
Total	54	100.00	39	100.00
Anemia (<10 gm %)	34	62.96	17	43.59

Z = 1.85, *P* < 0.05

The duration of gestation at which the delivery occurred could be assessed in 85 mothers of the 20-24 year age group, as the remaining 6 mothers did not have documented records of their last menstrual period. Preterm delivery occurred more frequently (*P*<0.001) among the teenage mothers. However, post-dated deliveries were slightly higher in the control group (10.59%) than in the study group (6.89%), though this was not significant. Yet, the number of term deliveries was significantly higher (*P*<0.005) in the control group [Table 3].

**Table 3:** Distribution of subjects according to duration of pregnancy at confinement

Duration of pregnancy in weeks	Study group		Control group	
		
	No.	%	No.	%
< 32	6	10.35	2	2.35
32-34	10	17.24	4	4.71
35-37	14	24.14	16	18.82
38-40*	23	39.66	37	43.53
41-42*	1	1.72	17	20.00
>42	4	6.89	9	10.59
Total	58	100.00	85	100.00
Term delivery*	24	41.38	54	63.53

* Z = 2.61, *P* < 0.005

The incidence of low birth weight was significantly higher (*P*<0.0001) in babies born to teenage mothers than in those born to older women [Table 4]. Not a single newborn was found to be above 3 kg at birth in the study group, while none were below 1.5 kg in the control group. The average birth weight was 2.63 kg in the study group and 2.74 kg in the control group. This difference was strongly significant at *P*<0.0001.

**Table 4:** Distribution of subjects according to birth weight of babies

Birth weight of babies (Kg)	Study group		Control group	
		
	No.	%	No.	%
< 1.5	3	5.17	0	-
1.5-1.9	1	1.73	1	1.10
2.0-2.4	34	58.62	23	25.27
2.5-3.0	20	34.48	58	63.74
>3.0	0	-	9	9.89
Total	58	100.00	91	100.00
Low birth weight	38	65.52	24	26.37

Low birth weight: Z= 4.73, *P*<0.0001, Mean birth weight: Study group = 2.63 kg, Control group = 2.74 kg, Z = 6.59, *P* < 0.0001

## Discussion

Teenage pregnancy is often referred to as ‘at-risk pregnancy’ and is of grave concern. Teenage women face a greater risk of obstetric complications than women in their twenties. The risks are greatest for the very poor who have worse diets and the least opportunity for prenatal care. Social problems like illiteracy, poverty, and low socio-economic conditions aggravate the situation. Under the economic conditions prevailing in rural India, coupled with poor utilization of health services, the problem of adolescent motherhood is linked with child survival and maternal mortality and morbidity.(11)

The age at marriage varies in different parts of India, according to different social customs and ethnic and religious groups. In rural areas, early marriage is perpetuated by traditional beliefs regarding preserving a girl's chastity and family needs to reduce expenditure.(12) Teenage pregnancy is therefore coming up as one of the most important social and public health problems. The practice of family planning is still very limited in this group. Most adolescent girls in rural areas being illiterate are not aware of family planning methods, and even if they are aware they do not have easy access to family planning services or fail to utilize them due to inhibitions or pressure to attain motherhood to satisfy their mothers-in-law or husbands.(1)

Across countries and cultures, women have been victims to social pressure and are often in a position to neither regulate their pregnancy nor make decisions regarding their reproductive performance. Husbands and mothers-in-law are the primary decision-makers. In many cases, this decision making structure appears to be driven by a woman's lack of economic independence. Even access to the most effective services is highly dependent on the involvement of influential family members.(13–15) As a result, early pregnancy and its complications continue to remain highly prevalent.

In India, 10.3% of the female population belongs to the age group of l5-19 years.(16) In 1997, the age specific fertility rate was found to be 52.5 live births per 1000 rural women aged between 15-19 years.(11) Over the years, there has not been much improvement in the country's scenario as reported by the National Family Health Surveys 1, 2, and 3 where the median age at first birth for women aged 25-49 years was observed to be 19.4, 19.3, and 19.8, respectively.(17) The prevalence of teenage pregnancy in the study population was 24.17%, which lies within the range observed in India, which varies from 3% to 52%.(4,16,18) Complications of pregnancy were observed to be more among adolescent mothers in community- as well as hospital-based studies. The incidence of still birth, preterm delivery, low birth weight, and complications during pregnancy and labor like toxemia of pregnancy, eclampsia, and cephalopelvic disproportion were more in teenagers.(19,20)

Anemia is a common complication of teenage pregnancy.(2) Chahande, *et al*. reported 72.6% of teenage pregnant women to be anemic.(19) Osbourne, *et al.* observed a highly significant increase in the incidence of anemia (*P*<0.001) in pregnant teenagers, 11.1% as compared with 5.2% in the 20-24 year old age group.(21) In this study, the finding was similar, though the incidence, as a whole, was higher in both the groups than that observed by Osbourne.(21) Other authors, however, failed to observe similarly.(9,10,22,23) A study conducted among mothers of various age groups showed that anemia was lower in teenage mothers (33%) in comparison with those who were 20-30 years old (62.1%) and those who were in the 31+ year old group (71%).(9) Pachauri, too, found a lower incidence of anemia among teenagers than in the control group.(22) Ghosh observed little difference in the incidence of anemia between the younger and older teenagers.(10)

Prematurity rates have been reported to be higher in teenage mothers than in the older group by many authors. Probable causes for the higher incidence of preterm labor may be anemia, malnutrition, pregnancy-induced hypertension, or lack of antenatal care.(9,10,23) Foreign authors also observed similarly.(21,24) In this study, the incidence of preterm delivery among the teenage mothers (51.51%) was double that in the control group (25.82%). Preterm delivery in teenagers in this study was much higher than that reported by the other Indian authors, which varied from 13.7% to 31%.(8,10,19,23)

The average birth weight was lower in the study group (2.36 kg) than in the control group (2.74 kg). The prevalence of low birth weight also was significantly higher in the study group. Some authors reported a higher incidence of low birth weight among babies born to teenage mothers.(8,19) Horon, *et al.* found no significant difference in birth weights between the two groups. He observed that factors known to vary with birth weight included socio-demographic and anthropometric characteristics of the mother, antepartum care, time of onset of labor, the length of gestation, and the sex of the infant. Maternal socio-demographic characteristics included race, marital status, and hospital ward status. Gestational age was the most important variable in predicting birth weight.(25) Efiong and Banjoko stated that the risk of low birth weight among teenage mothers was small and it could be further reduced by good antenatal care.(26)

Teenage pregnancy is thus seen to be a serious problem, giving rise to many complications. However, in the first paper published by Harris in recent English medical literature, with regard to pregnancy and labor in young primipara, it was stated that from a purely obstetric point of view, young mothers were not at special risk. On the contrary, 16 was said to be the most favorable age to have a baby.(27) But in our present social perspective, teenage pregnancy must be discouraged to reduce the population crisis and family size and its social, economic, and educational consequences, in addition to a reduction in the multiple complications that may occur in the young mother and her newborn baby. If early marriages cannot be discouraged, as the situation still prevails in rural areas of India, three steps can be taken for prevention of complications of adolescent pregnancy through enhanced Family Welfare measures:

- Delay marriage as much as possible
- Delay the first pregnancy
- Delay subsequent pregnancies

It is encouraging to observe that the number of teenage marriages have shown a slight decrease in the country, as reported by the three National Family Health Surveys, where the percentage of women aged 20-24 years old married by age 18 were 54.2, 50.0, and 44.5, respectively.(17)

## Conclusion

This study highlights that nearly one fourth of pregnancies occur in teenage women, who have significantly higher rates of complications. This may cause retardation of growth and development, and also deprive them of their childhood and education with resultant deterioration of the overall health of the nation. The time has come to focus on this problem. Education, nutritional support, and family planning, along with creating awareness among the community and also the school girls about the importance of delaying marriage, reproductive health, family life, and population education will definitely help in transforming today's adolescent girls into healthy and responsible women, giving birth to a healthy future generation.
